# The Microbial and Chemical Terroir of Agarwood in French Guiana

**DOI:** 10.1007/s00248-026-02824-0

**Published:** 2026-07-15

**Authors:** Kenji Maurice, Nicolas Baldovini, Alba Zaremski, Jérémie Damay, Romain Lehnebach, Yannick Estevez, Marc Ducousso

**Affiliations:** 1 AGAP Institut, Univ Montpellier, CIRAD, INRAE, IRD, SupAgro, Montpellier, France; 2 Institut de Chimie de Nice, UMR 7272, Université Côte d’Azur, Parc Valrose, 06108 Nice, France; 3 CIRAD, UPR BioWooEB, 34398 Montpellier, France; 4 BioWooEB, Université de Montpellier, CIRAD, 34398 Montpellier, France; 5 ECOFOG, AgroparisTech, Cirad, CNRS, INRAE, Univ. Guyane, Univ. Antilles, Pariacabo, BP 701, 97387 Kourou Cedex, Guyane Française France

**Keywords:** Aquilaria, Oud, Microbiome, Chemistry, Terroir

## Abstract

Agarwood is a highly valued aromatic resinous wood formed in *Aquilaria* species following stress or infection, yet the putative microbial drivers of its quality remain poorly understood, particularly outside its native range. In this study, we investigated the bacterial and fungal communities associated with agarwood produced from *Aquilaria crassna* Pierre ex Lecomte planted in French Guiana and examined their relationships with volatile chemical compounds relevant to agarwood fragrance. Using high‑throughput sequencing and comprehensive chemical profiling, we characterized microbial community composition and agarwood volatile profiles across multiple cultivation plots. Despite spatial variability in microbial assemblages, agarwood samples exhibited a conserved chemical signature dominated by chromone derivatives and sesquiterpenoids, indicating the presence of a stable chemical terroir under Guianese environmental conditions. Network analysis revealed numerous bacterial and fungal taxa significantly associated with key chemical classes, suggesting potential microbial contributions to agarwood chemical complexity through plant–microbe interactions or microbial metabolic activity, although causality remains to be established. Comparative analyses with commercial agarwood samples from South-East Asia and the Middle East revealed a distinct chemical profile for Guianese agarwood, highlighting the influence of geographic origin on agarwood quality and supporting an extension of the terroir concept to woody aromatic products. Overall, this study demonstrates that *Aquilaria* trees cultivated in French Guiana can produce high‑quality agarwood and provides new insights into the interplay between microbial communities and agarwood chemistry. These findings lay the groundwork for the development of locally adapted, microbiome‑informed strategies for sustainable agarwood production.

**Supplementary Information:**

The online version contains supplementary material available at https://doi.org/10.1007/s00248-026-02824-0.

## Introduction

Agarwood is a resinous wood formed following infection or injury in trees of the family Thymelaeaceae, which originate from the Indomalesian region. Increasing evidence suggests that microbial infection plays a key role in inducing resin production in *Aquilaria* species, although the underlying mechanisms remain only partially understood [1, 2]. Beyond its medicinal uses, agarwood has been burned as incense since antiquity in Asia and the Middle East, where it is regarded as one of the most precious natural fragrant materials. Agarwood is also distilled to produce agarwood oil, a highly valued ingredient in fine perfumery. The principal agarwood‑producing genera, *Aquilaria* and *Gyrinops*, are currently listed on the IUCN Red List due to excessive exploitation of wild populations [3]. The planting of these species for industrial agarwood production may alleviate pressure on wild populations, and most current trade now relies on planted trees inoculated with formulations often kept secret that enabled agarwood formation. Although the agarwood formation process in *Aquilaria* trees is not yet fully understood, it is widely accepted that resin production is triggered by natural or deliberate wounding followed by colonization by environmental microorganisms [1, 4]. Among these factors, stress plays a central role, as *Aquilaria* trees produce and accumulate secondary metabolites in response to injury [5, 6]. To produce agarwood in managed plantation, induction techniques imply either mechanical wounding of the trunk, the use of chemical inducers [7] but also microbial-based techniques using fungal inoculum [1, 8]. These latter methods are often based on inoculation by *Aquilaria* endophytic or soil fungi, promoting a more natural and environmentally friendly solution to improve productivity and quality. Agarwood microbiome is known to differ from that of non-infected wood, and has been reported to contain Actinomycetes such as *Nocardia* and *Streptomyces*, and fungi such as *Fusarium* and *Trichoderma* [9, 10]. Fungal inoculum thus often originates from the specific microbiome of infected *Aquilaria* trees or from the surrounding soil. However, choosing the appropriate microbial strains to induce a stress response of the tree enabling to produce the molecules of interest to obtain high quality agarwood remains a difficult task [11]. Moreover, while the current knowledge on agarwood formation and deliberate induction are progressing [12], major challenges still remain concerning the quality of the resulting agarwood.

The chemical composition of agarwood has been studied extensively and is characterized by the occurrence of two main families of specialized metabolites: sesquiterpenoids and 2-phenylethylchromone derivatives [13]. Both of these groups contribute to the olfactory properties of agarwood: sesquiterpenoids are generally sufficiently volatile to be intrinsically fragrant, and some of them are well known strong odorants [14]. In contrast, 2‑phenylethylchromones have higher molecular weights and therefore much lower volatility; although essentially odorless, they act as precursors of lighter aromatic compounds produced during thermal degradation when agarwood is burned. Nevertheless, the identity of the most important olfactory contributors remains debated, and no clear relationship has yet been established between odor quality and specific chemical markers. In general, identifying key odorants in aromatic raw materials is challenging due to complex synergistic interactions and the disproportionate influence of trace constituents. It is particularly true in the case of complex mixtures like agarwood oils or extracts containing a very large number of specific constituents with high molecular complexity, and also showing an important variability of composition from one origin to the other. The chemical complexity of agarwood is partly attributable to the contribution of bacteria and fungi during resin development [11]. Microorganisms are well known to influence the flavor profile, nutritional chemistry, and sensory properties of numerous biological products. A well-known example is the grapevine associated soil and endophytic microbiota, which contribute to the taste and quality of wine [15, 16]. The combined influence of biotic and abiotic factors shaping product flavor and quality is increasingly described by the concept of “microbial terroir” [17, 18]. This concept, mainly referring to wine grapes, pertains to the geographic microbial signature of soil, rhizosphere and endophytic fungi in grapes and vineyards [19] on the fermentation process [20, 21] and ultimately on the wine taste. The microbial terroir, beyond wine grapes have also been found to influence the flavor chemistry of mustard seeds, where rhizosphere taxa predicted glucosinolate content, responsible for the desired spicy and bitter flavor of mustard seeds [22]. In addition to abiotic parameters such as climate, pH, nutrient content and management practices, local microorganisms also have a role on the quality and specificity of food products. While endophytic microbial communities of *Aquilaria* influence the process of agarwood formation, their impact on the resulting agarwood quality and signature is yet to be determined, particularly in the concept of microbial terroir. Importantly, these microbial communities are not only involved during resin formation in living trees but may also persist in harvested wood and become active during downstream processing. This is particularly relevant as wood chips are often macerated in water prior to hydro-distillation to improve oil extraction yield and quality. Therefore, microorganisms are potentially involved in two key steps of the process; infection of living trees, and fermentation of wood chips before distillation. Indeed, microbial diversity during maceration [10] and microbial consortia [23] were found to improve agarwood oil quality. Local endophytic microbial communities of agarwood may thus have an influence on the maceration process, and could also be targeted for improving the resulting agarwood oil quality.

In French Guiana, the *Aquilaria* tree is considered as a cash crop by farmers in the communities of the villages of Regina and Cacao. These farmers, originally from Laos and with a long agricultural tradition [24] possess traditional knowledge and expertise in cultivating the *Aquilaria* tree. In French Guiana, they are working to establish a sustainable *Aquilaria* production sector. To support high quality agarwood production, it is necessary to take into account local fungal and bacterial communities, as well as to estimate the quality of *Aquilaria* wood that has been planted outside its natural range. In this context, the primary objective of this study was to characterize the microbiome and chemical composition of agarwood produced in French Guiana. Specifically, we (i) described the fungal and bacterial communities associated with agarwood, (ii) investigated their relationships with chemical constituents linked to agarwood scent, and (iii) assessed the effect of geographic location on agarwood quality. We hypothesized that, as observed in other systems, local microbial communities contribute to variation in agarwood chemical profiles, supporting a “microbial terroir” concept that we extend here to a broader framework of “chemical terroir”.

## Material and Methods

### Study Site

**Fig. 1:**
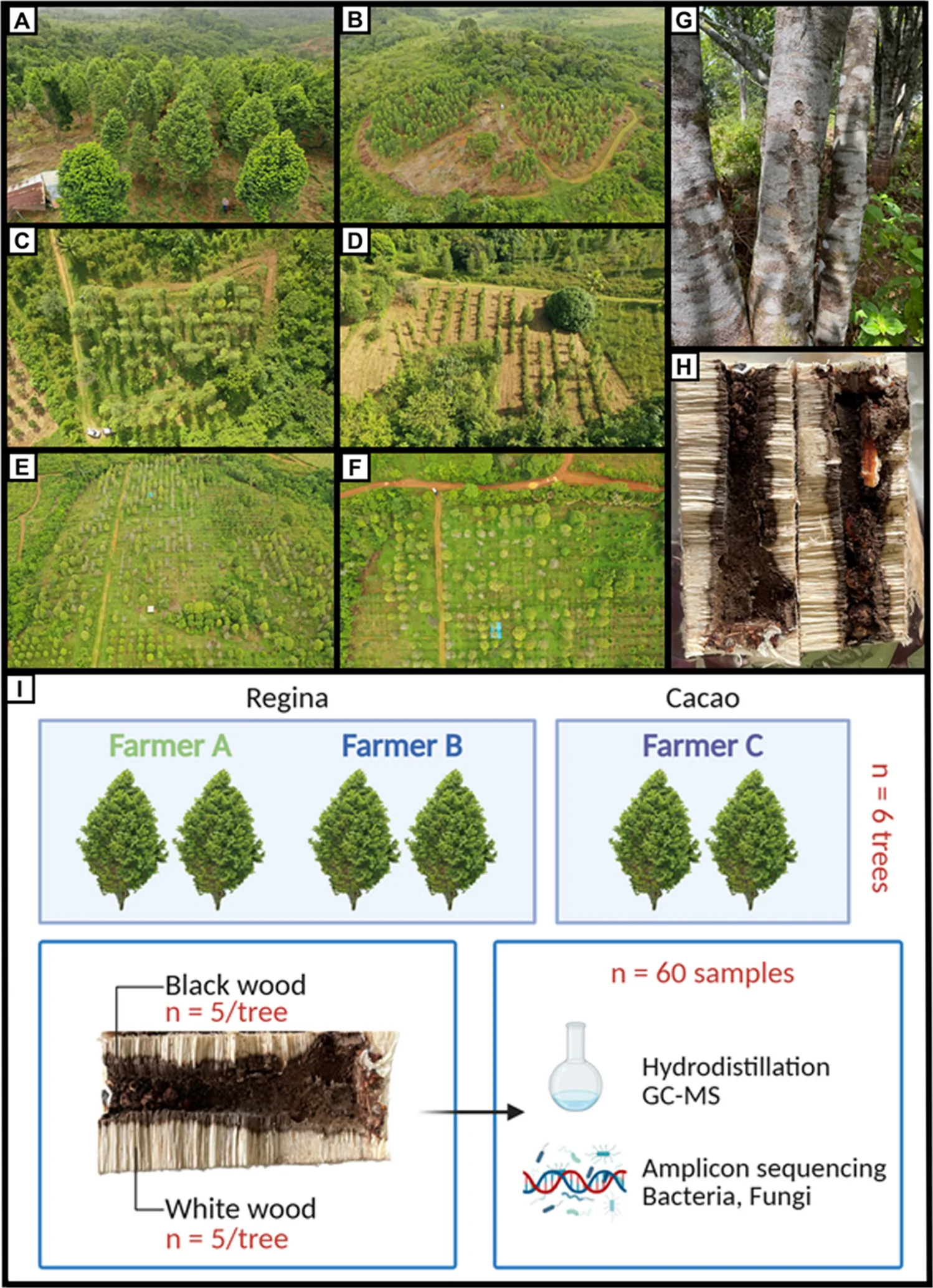
Aerial views of sampling sites in experimental *Aquilaria crassna* plantations. Farmers A (**A**, **B**) and B (**C**, **D**) are located in the commune of Regina, while farmer C (**E**, **F**) is located in the commune of Cacao, in French Guiana. Previous tree inoculation is shown in **G**, and a cross-section at the inoculation point revealing blackwood is shown in **H**. The sampling design is described in **I**, where two *Aquilaria crassna* were sampled in each plot. Black wood and White wood samples were collected from five trunk cross-sections of each tree for a total of 60 samples for chemical and microbial analysis (*n* = 30 white wood, *n* = 30 black wood)

Sampling was conducted in two agricultural plots located in the Regina (Farmer A: 4°17′42.3"N 52°10′55.2"W; Farmer B: 4°17′41.8"N 52°10′54.6"W) and one in Cacao (Farmer C: 4°32′22.4"N 52°28′38.7"W) villages in French Guiana. At each of the three sites, two *Aquilaria crassna* trees previously inoculated in November 2022 with three fungal strains (*Antrodia vaillantii* (DC. Ryvarden), *Pycnoporus sanguineus* (L.) Murril, and *Coriolopsis polyzona* (Pers.) Ryvarden) were selected. Inoculation was performed using fungal strains grown on Modified Melin-Norkrans medium, mixed with sterile water, and injected in freshly 10 mm drilled holes in the trunk. Given that agarwood formation following inoculation is highly localized within the tree, the sampling strategy was designed to capture intra-individual variability. For each tree, the trunk was sectioned at the inoculation point, and five cross-sections were collected. From each cross-section, two samples were taken, corresponding to blackwood (resinous zone) and whitewood (non-resinous zone), resulting in a total of 60 samples (Fig. 1I). These cross-sections represent pseudo-replicates within individual trees and were used to assess fine-scale heterogeneity in microbial communities and chemical composition. Details of the sampling sites, inoculated trees, and extent of blackwood formation are presented in Fig. 1A–H.

### Soil Sampling and Analysis

Five soil samples were collected at a depth of 20 cm at each sampling site, in close proximity to Aquilaria trees and in areas free of plant litter. Soil samples were sieved at 2 mm and dried at 105 °C for 48 h prior to analysis, resulting in a total of 15 samples. The study focused on elemental composition and pH as primary descriptors of soil geochemistry potentially influencing agarwood formation. While additional parameters such as carbon and nitrogen content or soil moisture were not measured, these variables are often less spatially discriminant at the scale of the studied sites compared to trace and major elemental composition. The latter was therefore prioritized to capture potential site-specific geochemical differences. To measure the elemental composition of each soil, samples were processed in triplicate using X-ray fluorescence spectroscopy (XRF). For each sample, triplicates were pressed at a 20 tons pressure for 2 min with 1:3 (v:v) of SpectroBlend® (SCP Science). Three technical measures on each triplicate, using the ‘geoexploration’ calibration with a total exposure time of 105 s on the XRF S1 Titan analyzer (Bruker) was used to measure the soil atomic composition of elements from magnesium to uranium, leading to nine measurements for each soil sample. The soil pH was measured at a 1:5 (v:v) ratio of H_2_O using a pH meter (pH Meter Knick 766, Knick International). Soil composition was visualized using composition barplots for the main elements (Al, Ca, Co, Cr, Cu, Fe, K, Mg, Mn, Si and Ti). Principal component analysis (PCA) using centered log-ratio transformed abundances were used to visualize soil composition by farmer plots.

### Library Preparation and Sequencing

Wood chips were crushed in liquid nitrogen using a sterile mortar and pestle. Approximatively 100–150 mg of wood were then used for DNA extraction using the FastDNA Spin Kit for Soil (MP Biomedicals™). DNA concentrations were measured by fluorescence with a Quant-iT™ PicoGreen™ dsDNA Assay Kit (ThermoFisher Scientific™, Waltham, Massachusetts, USA) using a Tecan Spark ® (Tecan Group Ltd., Mannedorf, Switzerland) and normalized to 1 ng.μL^−1^ before PCR amplification. Primers 479 F (5′ CAGCMGCYGCNGTAANAC 3′) and R888 (5′ CCGYCAATTCMTTTRAGT 3′) were used to amplify the V3-V4 regions of the 16S rRNA gene [25]. Primers ITS86F (5′ GTGAATCATCGAATCTTTGAA 3′) and ITS4 (5′ TCCTCCGCTTATTGATATGC 3′) were used to amplify the fungal ITS2 [26]. Reactions were performed in 20 μL and consisted of 10 μL of Buffer Master mix containing Thermo Scientific Phusion™ HighFidelity DNA Polymerase (ThermoFisher Scientific™), 0.5 μL of DMSO, 4.5 μL of DNAse free water, 3 μL of DNA (0.3 ng. μL-1), 1 μL of forward primer and 1 μL of reverse primer tagged with a short nucleotide sequence. The PCR conditions used were identical for the fungal and bacterial amplifications and were performed in a Veriti 96-well Thermal Cycler (ThermoFisher Scientific™) under the following conditions: 10 min initial denaturation, 35 cycles each including denaturation at 94 °C for 10 s, annealing at 55 °C for 20 s, and extension at 72 °C for 20 s, with a final polymerization extension at 72 °C for 7 min. All reactions were performed in triplicate and then pooled before deposition on 2% agarose gel, with the corresponding negative control, for amplification quality control. Amplification products of ITS2 region were purified using Agencourt AMPure XP beads (Beckman Coulter Inc., Fullerton, California, USA) and performed on a magnetic rack with a 1:1 (v:v) ratio of AMPure XP per PCR product. The magnetic beads were then rinsed twice with 70% ethanol before final elution in 70 μL of Qiagen EB buffer. For 16S purification, an additional PCR product migration step on 2% agarose gel (80 V, 90 min) was performed to separate and recover the band specific to bacterial 16S (lower band, around 400 bp) and eliminate plant chloroplastic 16S DNA (higher band, > 600 bp, co-amplified during PCR). The recovered bands were purified using the QIAquick gel purification kit (Qiagen, Hilden, Germany). The purified PCR product concentration was then measured with PicoGreen and one equimolar pool (50 ng/sample) for each marker gene (16S and ITS2) was prepared. Each pool was finally purified twice using Agencourt AMPure XP beads, quantified and finally eluted in 40 μL of EB buffer (Qiagen). Library sequencing was performed on a 2× 250 bp MiSeq system (Fasteris SA, Switzerland).

For taxonomic assignment of fungi, OTUs were constructed on a 97% ITS clustering, while ASVs were used for bacteria (16S). We used a pipeline based on VSEARCH 2.18.0 [27] and available in GitHub (https://github.com/BPerezLamarque/Scripts/) for data processing. Briefly, only paired-end reads were retained and then merged using the fastq_mergepairs function with default parameters. We applied a quality check and removed merged reads with more than two alignment errors. Then, filtered merged reads were assigned to the respective samples based on tagged primers (demultiplexing) with 0 accepted error regarding the primers and tag sequences using Cutadapt 3.5. We removed chimeras de novo by using the VSEARCH uchime3_denovo option and assigned the taxonomy to each amplicon sequence variant (ASV) and Operational Taxonomic Unit (OTU) using the usearch_global option with default parameters. Taxonomic annotation was performed with SILVA (v.138.2) database for bacteria (16S rRNA) and UNITE database (v.9.0) for fungi (ITS). For bacteria we used ASV (16S rRNA) while 97% OTUs were used for fungi (ITS), as recommended by Tedersoo et al*. *[28]. It should be noted that taxonomic resolution may be constrained by both the targeted barcode regions and the completeness of reference databases. Consequently, some ASVs/OTUs could only be assigned to higher taxonomic levels or remained unclassified. These limitations affect taxonomic annotation rather than sequence inference itself. The ASV and OTU tables were then filtered from contaminants based on comparisons with DNA extraction negative controls using the DECONTAM (prevalence method) R package [29]. Only ASVs and OTUs with long sequences (> 200 bp), assigned to the bacterial or fungal kingdom, presenting an acceptable abundance (≥ 10 reads in total dataset) and amplified in at least one sample, were ke0pt for subsequent analyses. ASV and taxonomic tables are provided in Supplemental Tables 1–4, with the associated samples metadata in Supplemental Table 5.

### Characterization of Volatile Constituents in Wood Samples: Sample Preparation

To determine the chemical composition of the volatile fraction of the same samples used for microbiome analysis, we performed their hydrodistillation using a modified glass made Clevenger apparatus (Modverre, Grasse, France, Supplemental Fig. 1). This approach was selected to enable direct comparison between microbial communities and the chemical profiles of agarwood, which is central to the objectives of this study. Hydrodistillation allows the extraction of the volatile fraction corresponding to agarwood oil, the second main end product of the agarwood industry after wood chips, and which can be directly analyzed by direct injection in a gas chromatography-mass spectrometry (GC–MS) system. Therefore, this method provides a relevant proxy for the composition of agarwood oils that would be obtained by the large-scale industrial distillation of similar wood samples. Even if the obtained extract cannot be considered as an essential oil *stricto *sensu [30], it is nevertheless constitutes a suitable model for analytical investigation on industrial agarwood oil composition. However, in the context of the present work, which involves small sample amounts (< 1 g), a classical Clevenger apparatus is not adapted, as the low quantity of oil produced (few milligrams) would be lost in the hydrolate. We therefore used a modified system specifically designed to recover trace amounts by trapping the organic volatiles in a solvent (tert-butyl methyl ether) within a cooled double jacketed collection tube to prevent the evaporation of the solvent during hydrodistillation. After optimization, the following parameters were applied to all hydrodistillations: the powdered wood sample (100–600 mg) and 40 ml water were placed in a 100 ml round bottomed flask equipped with an ovoid stir bar and connected to the Clevenger system. Two ml of tert-butyl methyl ether were added in the refrigerated collecting tube previously filled with water. The mixture was heated in an oil bath (145 °C) under stirring for 19 h. At the end of the distillation, the solvent phase was collected, and concentrated until *ca.* 0.2–0.3 ml by flowing a gentle stream of argon, then diluted in *ca*. 1 ml of dichloromethane. The extract was dried by filtration through a short pad of magnesium sulfate in a Pasteur pipette, and spiked with ca. 100 mg of a carefully weighed 0.02% solution of tetradecane in toluene (internal reference). It was then concentrated under a flow argon and finally adjusted to a volume of *ca*. 50–100 μl by adding dichloromethane.

### Characterization of Volatile Constituents in Wood Samples: GC–MS/FID Analyses

Gas chromatography—Mass spectrometry/Flame Ionisation Detector (GC–MS/FID) analyses were carried out using an Agilent 6890 N gas chromatograph equipped a fused silica capillary column DB-1MS (30 m × 0.25 mm i.d., film thickness: 0.25 µm, J&W122-0132). The analytical parameters were the following: the carrier gas was helium at a flow rate of 2 mL.min⁻^1^, the oven temperature was programmed from 60 to 280 °C at 3 °C/min and completed by a post run (300 °C, 5 min). Five μl samples of the concentrated extract prepared as described above were injected in splitless mode and the injector temperature was 250 °C. The column flow was split using a G3184-60065 splitter (Agilent), and transferred to a FID detector (250 °C, hydrogen and air flows at 40 and 450 ml/min, respectively) and to an Agilent 5973 N mass selective detector working in electron impact (EI) mode at 70 eV (scanning over 40–450 amu range in SCAN mode). The temperatures of the MS ion source and transfer line were 230 and 280 °C, respectively. Linear retention indices (LRI) were determined from the retention times of a series of *n*-alkanes with linear interpolation. The main constituents were identified by comparison of their mass spectra and LRI with those of pure compounds registered in commercial libraries and literature data, and with a laboratory-made database built from authentic compounds, with the help of SearchReview software (https://waleson.eu/). The absolute amount of the constituents was determined by GC-FID using predicted response factors as previously reported [31]. To position the chemical composition of Guianese agarwood within the range of commercially available products, samples of commercial blackwood purchased from street markets in Riyadh and AlUla (Saudi Arabia), likely originating from the Indomalayan region, were hydrodistilled and analyzed using the same protocol. These samples were used as comparative references rather than as strictly controlled representatives of their geographic origin. The chemical abundance and compounds assignments are provided in Tables 6, 7.

### Microbial and Chemical Diversity

To assess if sequencing depth was sufficient to retrieve microbial abundance, rarefaction curves were performed (Supplemental Fig. 2) using the mirlyn package [32]. Relative abundances of bacterial and fungal community and chemical compositions were then visualized using bubble plots at the genus level according to the wood type and farmer plots. To assess the differences in microbial diversity in black and white wood samples and as a function of sampling sites, Hill diversity index for three orders of diversity (q = 0, 1, and 2) were calculated and the significances of their differences assessed using Anova followed by Tukey HSD tests.

**Fig. 2:**
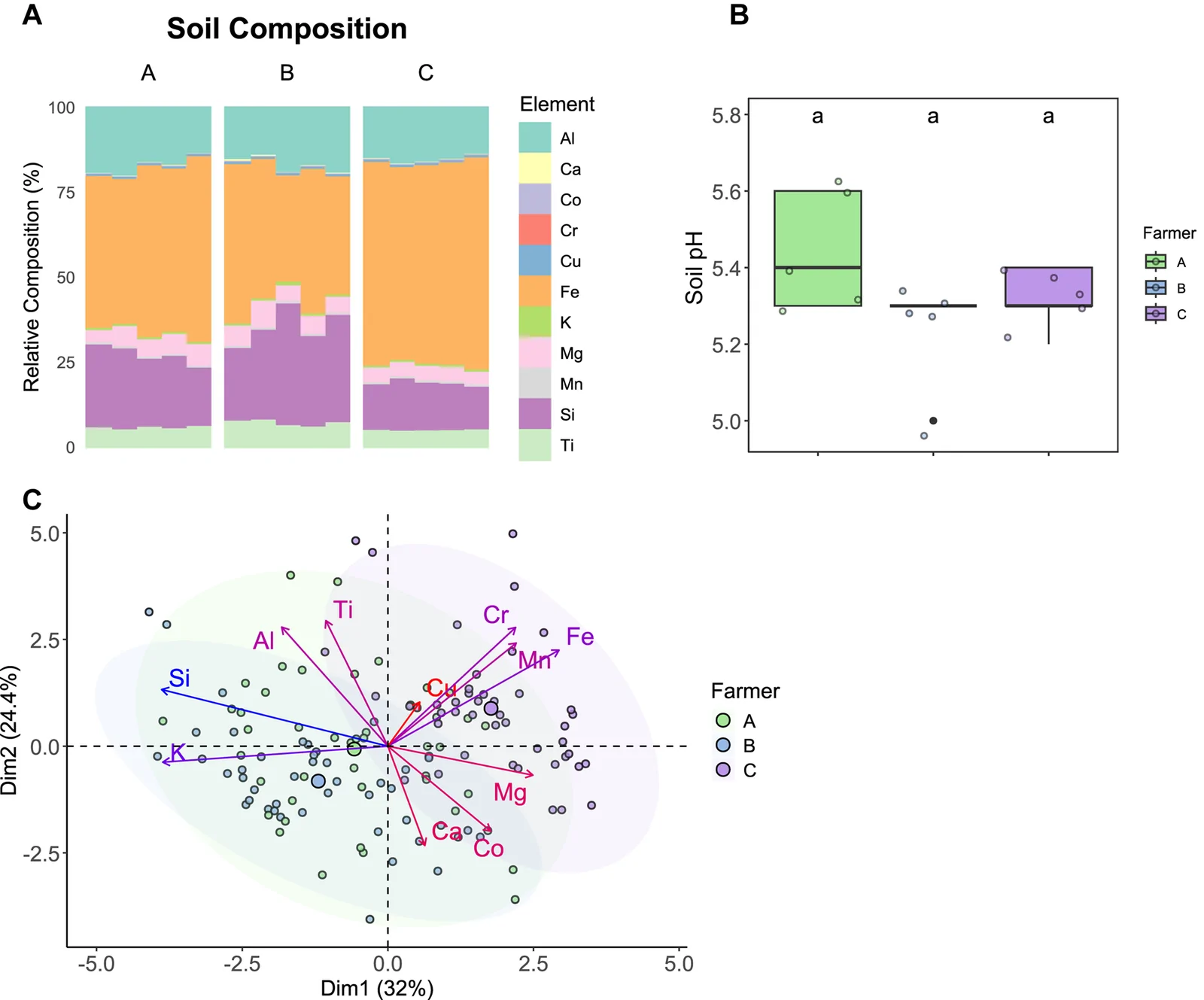
Soil composition for each farmer plot (**A**) and the associated pH (**B**). Similar letters represent no significant differences assessed using Anova followed by Tukey HSD post-hoc test. Principal component analysis of the soil composition according to the farmer plot, dimension 1 explain 32% of the variance and dimension 2, 24.4%. Vectors represent elements and are colored according to their contribution (**C**)

### Microbial and Chemical Composition

To evaluate how microbial communities vary with wood type and geographic origin (farmer plots), and how these variations relate to wood chemistry, we used complementary multivariate approaches aligned with three main objectives: (i) test the influence of wood type and location on microbial and chemical composition, (ii) identify key discriminant features, and (iii) evaluate the strength of association between microbial communities and agarwood chemical profiles.

First, to test the effects of wood type and geographic origin on microbial community structure, Hellinger-transformed microbial abundances and clr-transformed chemical abundance were analyzed using redundancy analysis (RDA). The proportion of explained variance (R^2^) and its significance were assessed using PERMANOVA (Bray–Curtis distance) with the model: microbial abundance ~ wood type × farmer. For chemical composition, clr-transformed data were analyzed 

using non-metric multidimensional scaling (nMDS), with significance tested by PERMANOVA. Comparisons between Guianese and commercial agarwood samples were also performed using nMDS to position our samples within the broader chemical diversity.

Second, to identify microbial taxa and chemical compounds associated with wood type (white vs black wood), differential abundance analyses were conducted using DESeq2 [33]. Log2 fold changes (Log2FC) and associated p-values were used to identify discriminant features. Microbial and chemical datasets were analyzed separately.

Third, to quantify within-group heterogeneity and assess the relationship between microbial and chemical variability, dispersion analyses were conducted using Aitchison (clr-based) distances. Differences in dispersion between wood types were tested using betadisper and permutest. The relationship between microbial and chemical variability was then evaluated using linear models of microbiological dispersion (ITS or 16S), including wood type and its interaction with chemical dispersion. To further assess concordance between datasets, Spearman ρ correlations, Mantel tests, and Procrustes analyses were performed on ordinated distance matrices.

### Co-Occurrence Network Analysis of Microbial-Chemistry

In order to accurately assess the correlations between microbial taxa and volatile constituents, network analyses based on covariance relationships were carried out. All the constituents were classified according to their putative biosynthetic origin to facilitate the statistical treatment of the chemical information. Among all the volatile constituents, sesquiterpenoids were easily recognized and associated to seven subgroups defined by their sesquiterpenic skeleton (bisabolane, eremophillane, eudesmane, guaiane, humulane, vetispirane, zizaane). Not surprisingly in view of the classical composition of agarwood oils, many aromatic compounds were also identified, and were classified either as “chromone derivatives” or “possible chromone derivatives”, depending on the degree of confidence we had on their suspected biogenetic origin. In addition to these groups, the other families were “linear/fatty acid derivatives”, “adulterants” and “possible pollutants” (to distinguish respectively suspected voluntarily and unvoluntary added compounds, mostly in commercial samples). Moreover, many minor compounds were not present in our database and could not be identified with certainty, therefore they were classified as “unidentified”. Considering microbial and chemical datasets as compositional and the paired samples design of our study, we used the cross-kingdom network inference using SPIEC-EASI [34, 35], which is robust to the bias of using two compositional datasets [36]. Multi domain SPIEC-EASI was thus used to build co-occurrence networks for agarwood samples (black wood) using the Meinshausen-Buhlmann's neighborhood selection method using 100 repetitions and 100 subsamples for the Stability Approach to Regularization Selection (StARS) algorithm [37]. The optimal coefficient matrix and symmetric matrix with edge-wise stability was then retrieved after verification of the optimal network stability at the selected sparsity. Sub-networks according to each chemical class were then created using the original ITS-chemistry and 16S-chemistry cross-kingdom networks. To do so, for each chemical class, we retrieved chemical nodes (named hereafter ‘core nodes’) belonging to each chemical class as well as their connections to other chemical compounds and microbial taxa. The internal microbial edges were removed (bacteria-bacteria or fungi-fungi edges) to specifically assess the interaction of microbial taxa and chemical compounds. Then, we calculated the number of positive and negative edges, as well as their taxonomic assignation (chemical or microbial class). Finally, to identify the significant covariance relationships between each microbial class and genus to each chemical class, the ratio of positive to negative edges were calculated for each sub-network specific of each chemical class. Its value (positive, negative or neutral) is an indication of the overall link between microbial taxa and chemical compounds found in agarwood.

## Results

### A Ferralitic Tropical Soil

All plantations were established on ferralitic tropical soils characterized by high iron concentrations (50.5 ± 6.1%; 41.2 ± 7.6%; 60.9 ± 4.3% for farmer A, B and C respectively), followed by Si (20.2 ± 5.0%; 27.4 ± 6.9%; 13.7 ± 2.7%), Al (16.1 ± 4.1%; 16.4 ± 3.6%; 14.9 ± 3.0%), Mg (6.9 ± 4.0%; 6.6 ± 3.6%; 5.1 ± 1.9%) and Ti (5.5 ± 0.4%; 7.0 ± 1.0%; 4.7 ± 0.2% for farmer A, B and C respectively). Soil pH did not differ significantly among plantations (mean pH = 5.3 ± 0.1, *p*.value = 0.0786). Relative abundances of each element for farmer plot are available in Supplemental Table 8. The soil from farmer C plantations had a higher Fe, Cr and Mn content, and a lower K and Si content compared to the other plantations (Supplemental Fig. 3), resulting in a contrasted soil composition compared to the other plantations (Fig. 2C).

**Fig. 3:**
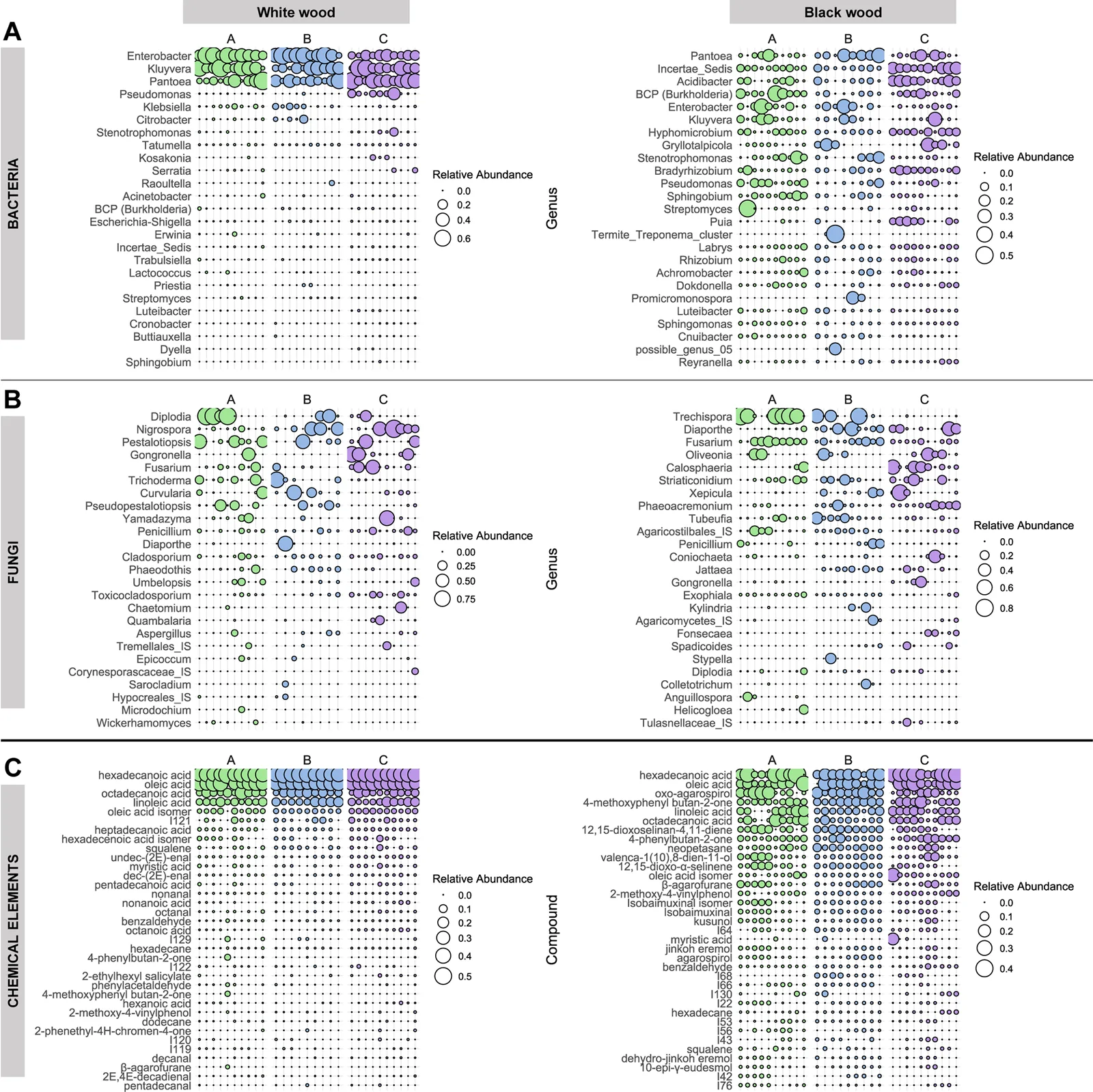
Bacteria (**A**) and Fungi (**B**) community composition (genus level) for each sample at each site (Farmer A, B and C), ordered by relative abundance and facetted by wood type (white or black). Each column corresponds to a wood sample, and the size of the bubbles is proportional to the relative abundance of the taxonomic level in line. Chemical composition of the wood volatiles compounds samples is also presented (C). Only the top 25 taxa and chemical compounds in relative abundance are presented

### Agarwood Microbial and Chemical Composition

*Enterobacter*, *Kluyvera* and *Pantoea* were the main bacterial genera found in white wood and this assembly did not vary significantly according to sampling sites (Fig. 3.A). *Diplodia*, *Nigrospora*, *Pestalotiopsis*, *Gongronella*, *Fusarium*, *Trichoderma* and *Curvularia* were the main fungal genera found in white wood (Fig. 3B). High concentration of fatty acids (hexadecanoic, oleic, octadecanoic and linoleic acids) were found in white wood samples, and their abundance did not significantly vary between samples nor sampling sites (Fig. 3C). On the other hand, *Pantoea*, an unknown genus, *Acidibacter*, *Burkholderia* and *Enrotobacter* were the main bacterial genera found in black wood (Fig. 3A) and their relative abundance significantly vary between samples, and to a higher extent, between sampling sites. *Trechispora*, *Diaporthe*, *Fusarium*, *Oliveonia*, *Calosphaeria* and *Striaticonidium* were the main fungal genera (Fig. 3B), their abundance also significantly varies according to samples and sampling sites. Black wood volatile constituents were generally more abundant, their number was also higher and their chemical structures were more diverse. Even if the fatty acids abundant in white wood were still present in black wood, we also found high amounts of typical agarwood compounds [38] such as oxo-agarospirol, 4-methoxyphenyl-butan-2-one, β-agarofurane, jinkoh eremol, agarospirol and dihydrokaranone (Fig. 3C). These microbial and chemical patterns were confirmed by the differential abundance analysis, where a high share of *Pseudomonadota* bacteria was found specific to the white wood, while a higher diversity of bacteria, belonging to diverse phyla was specific to the black wood (Supplemental Fig. 4A; Supplemental Table 9). This was also the case for fungi, where all taxa differentially abundant in white wood belonged to Ascomycota phyla, such as *Curvularia*, *Nigrospora* or *Chaetomium*. In the black wood, both Ascomycota, Oomycota and Basidiomycota were differentially abundant. The proportion of sequences that could not be assigned at the genus level represented a small fraction of the communities, accounting for 3.79 ± 6.17% in bacteria and 4.39 ± 6.60% in fungi. Interestingly, a high share of unidentified fungal species (Incertidae Sedis = IS) was found specific to the black wood (Supplemental Fig. 4B). Linear and fatty acids were found highly specific to the white wood, while a more diverse chemical diversity was specific to the black wood (Supplemental Fig. 4C). We found a specific signature of French Guiana agarwood samples when compared to foreign samples from South-East Asia (Figure S5A). Chromone derivatives, possible chromone derivatives, and sesquiterpenoids were more abundant in Guiana agarwood samples (Supplemental Fig. 5B). In addition, compounds such as di-n-butyl phthalate were detected specifically in commercial samples. While such compounds are sometimes associated with intentional adulteration (e.g., use as fixatives or solvents in perfumery products) [39], alternative explanations cannot be excluded. These include contamination during storage, handling, or transport (e.g., contact with plastic materials). Therefore, these signals should be interpreted cautiously and not unambiguously attributed to deliberate adulteration.

**Fig. 4:**
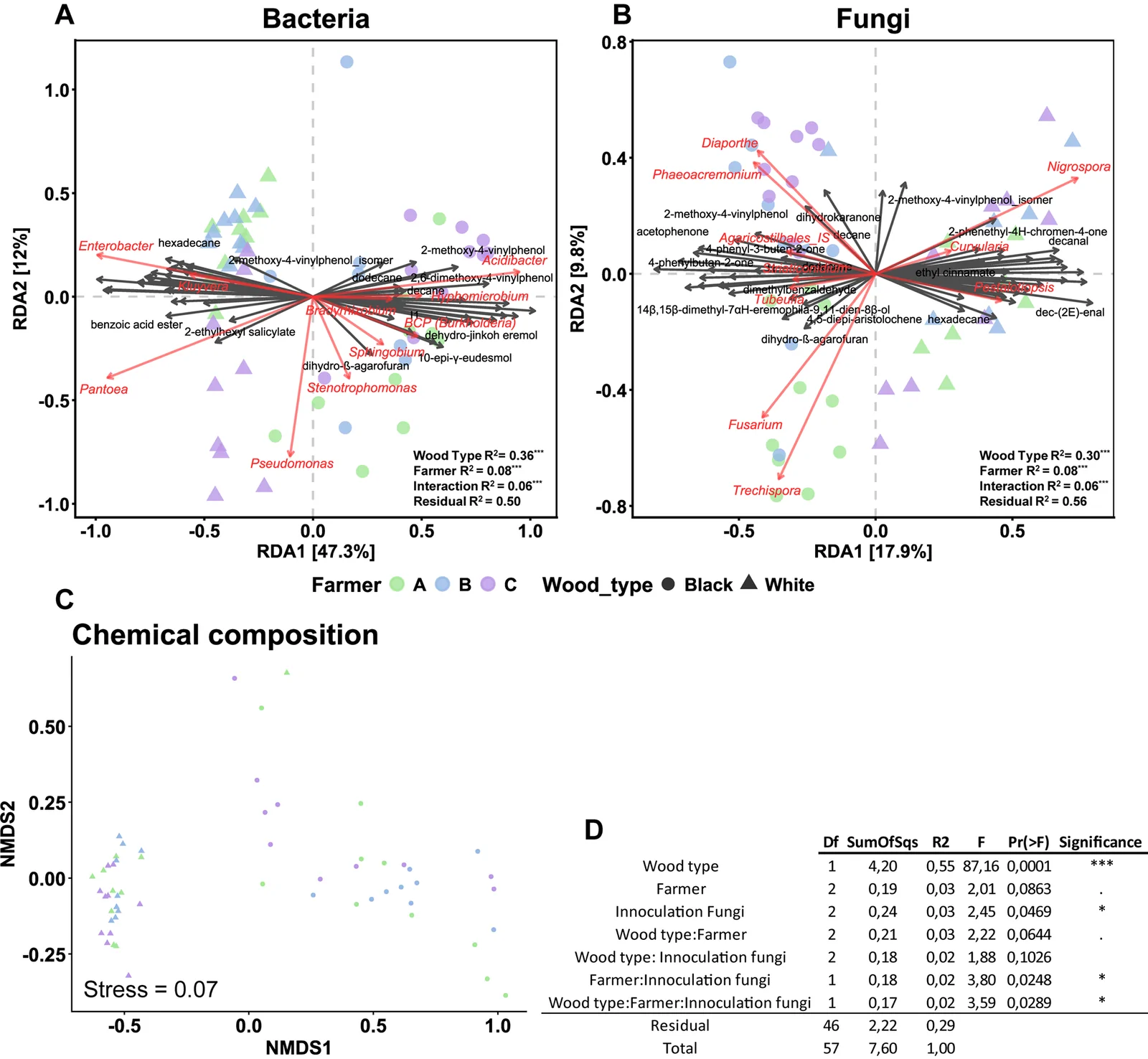
Redundancy Analysis of Bacteria (**A**) and Fungi (**B**). Black arrows represent chemical compounds variables, while red arrows denote microbial genus. Points are color-coded by site, while their shape represent wood type. The explained variance for dimensions 1 and 2 is displayed on each panel as percentages. Explained variance of each factor terms and their interaction on the bacterial and fungal community composition are also presented with their associated significance. nMDS of the chemical compounds based on their Hellinger-transformed relative abundances (**C**). The associated Permanova is also presented in the table D

**Fig. 5:**
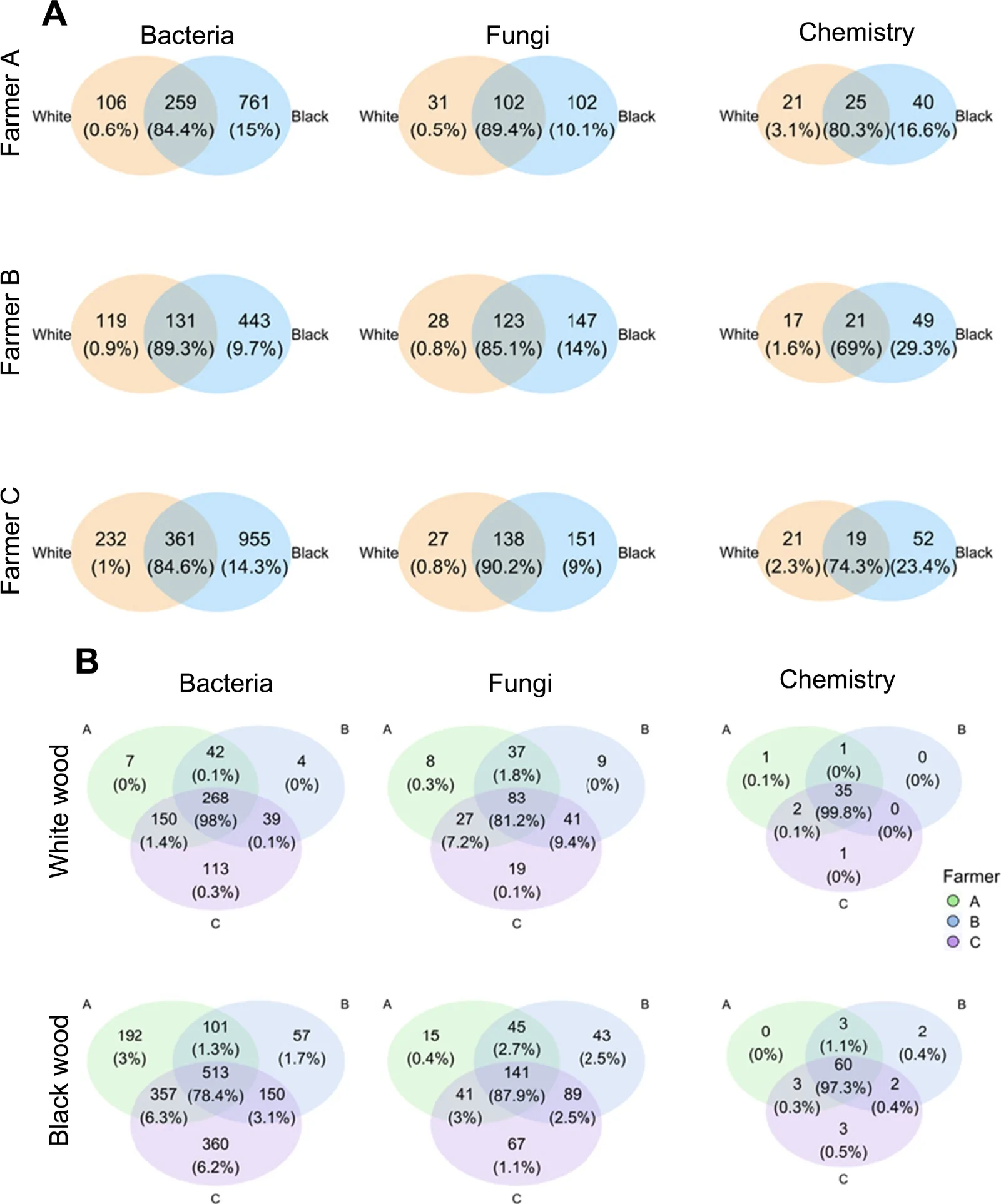
Venn diagrams showing the unique and shared Bacteria ASVs, fungal OTUs (counts and sequence percentages), and chemical compounds. Comparisons between whitewood and blackwood for each farmer (**A**). Comparisons between farmers within each wood type (**B**)

### Inter- and Intra-sample Variability of the Microbial and Chemical Composition

Overall, dispersion varied between black and white woods for chemistry and microbial communities (Supplemental Fig. 6A). Chemical composition dispersion was a strong positive predictor of microbiological communities’ dispersion (ITS: F₁,₄₉ = 21.18, *p* = < 0.001; 16S: F₁,₄₉ = 206.49, *p* < 0.001), with an effect of wood type on bacterial dispersion (16S: F₁,₄₉ = 107.58, *p* < 0.001), but no interaction between chemical dispersion and wood type. In standardized models, the chemistry slopes remained significantly positive (ITS β = 0.410, *p* = 0.043; 16S β = 0.195, *p* = 0.034), and white wood showed lower dispersion than black at average chemistry (ITS β = − 0.769, *p* = 0.035; 16S β = − 1.578, *p* = 3.01e − 10; Supplemental Fig. 6B). Model fit was moderate for ITS (Adj. R^2^ = 0.31) and high for 16S (Adj. R^2^ = 0.86).

**Fig. 6:**
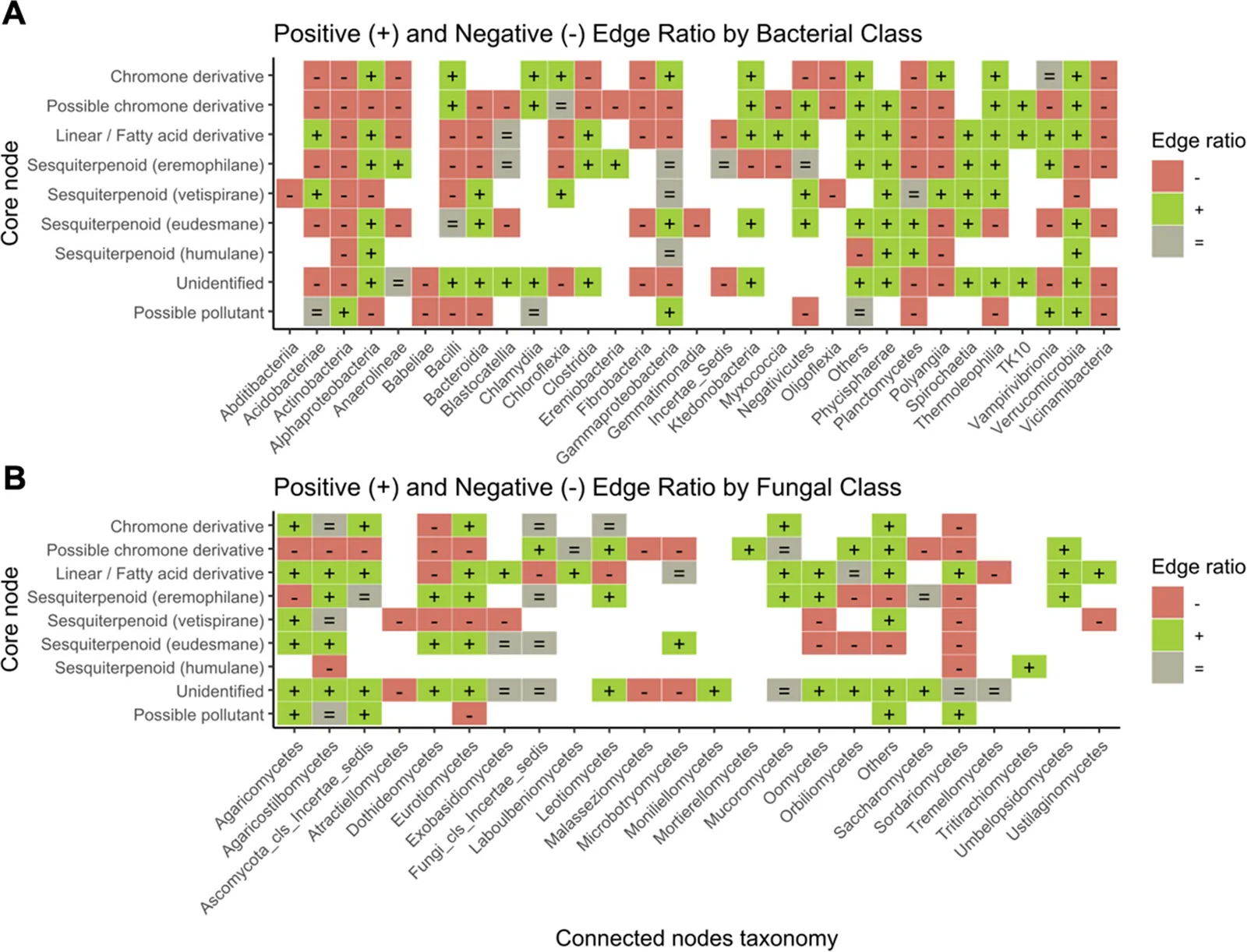
Ratio of positive to negative significant edges in each chemical compound class based on bacterial (**A**) and fungal (**B**) class. A negative edge ratio (-, in red), means more negative to positive edges are found for each microbial class to the relative chemical class, a positive edge ratio (+, in green), means more positive to negative edges are found for each microbial class to the relative chemical class. Equal ratio of positive to negative edges are also reported (=, in grey). Only significant edges ratio are reported

Overall sample-to-sample correlations between chemical and microbiological dispersion were moderate for fungi (Spearman ρ = 0.434, *p* = 0.001) and strong for bacteria (ρ = 0.746, *p* < 2.2e − 16). Mantel tests confirmed significant correspondence between chemical and microbiological distance matrices (chemistry–ITS: r = 0.418, *p* = 1e − 04; chemistry –16S: r = 0.390, *p* = 1e − 04). Ordination structures aligned well between chemistry and microbiology (Procrustes correlations: chem–ITS r = 0.677, *p* = 1e − 04; chem–16S r = 0.720, *p* = 1e − 04), supporting substantial shared multivariate patterns (Supplemental Fig. 6C). Chemical composition strongly structures both bacterial and fungal communities, with bacteria exhibiting more deterministic, chemistry-driven assembly than fungi.

### The Terroir Effect on Microbial and Chemical Composition

Concerning the overall community structure, wood endophytic bacterial beta diversity varied strongly according to the wood type (Fig. 4A; R^2^ = 0.36), while farming plot had a limited effect (R^2^ = 0.08). This pattern was similar for fungi, where wood type had the major effect on beta diversity (Fig. 4B; R^2^ = 0.30), while farming plot had a limited effect (R^2^ = 0.08). Redundancy analysis (Fig. 4C) also explained a higher shared of bacterial community composition (Dim 1 = 47.3% and Dim 2 = 12%) than for fungi (Dim 1 = 17.9% and Dim 2 = 9.8%).

Concerning the specificity of sequences and taxa found in *Aquilaria* wood, a high percentage of sequences were shared between black and white wood samples for all farmer plots (Fig. 5A), ranging from 84.4 to 90.2% of shared sequences. A higher share of unique sequences was found in black wood compared to white wood for both fungi and bacteria, where 9 to 15% of sequences were unique to black wood, while only 0.5 to 1% of sequences were unique to white wood. Conversely, a high number of taxa were found specific to black wood samples, where up to 955 bacterial taxa (out of 1548) were specific to black wood samples for the farmer C plot. This high diversity and high specificity of bacterial taxa in black wood samples was conserved for all plots. For fungi, the share of black wood specific taxa was similar to that of shared taxa. Chemical compounds also had a high proportion of common compounds between black and white woods, but a high number and proportion was specific to black wood (40 to 52 compounds). Concerning the specificity of microbial structure based on sampling sites, while a high share of sequences was found in samples from all locations (98.0% and 81.2% for bacteria and fungi of the white wood; 78.4% and 87.9% for the black wood), a high number of taxa were not found in all plots (Fig. 5B). For example, the farming plot C had a high number of specific taxa, both in white (113 out of 623 bacterial ASV) and black wood (360 out of 1 730 bacterial ASV). However, between the three sampling sites, the chemistry of white and black wood remained highly consistent (99.8 and 97.3% of share compounds respectively).

### Association between Microbial Members and Chemical Composition

The resulting sub-networks of chemical class and microbial composition revealed that each chemical class had specific relationships with different microbial taxa. Linear fatty acid derivatives had the highest connections with bacterial taxa (Supplemental Figure S7, Supplemental Table 10); core degree = 68.4), followed by possible chromone derivative (core degree = 66.2), while sesquiterpenoid humulane had the lowest (core degree = 38). The pattern was similar for fungi, where possible chromone derivative had the highest connection with fungal taxa (Supplemental Figure S8; core degree = 15.2), followed by linear fatty acid derivative (core degree = 13.8), and sesquiterpenoid humulane having the lowest (core degree = 9). The most connected bacterial nodes to chemical compositions belonged to the Alphaproteobacteria, Gammaproteobacteria, Actinobacteria and Planctomycetes bacterial class (Supplemental Fig. 9.A; Supplemental Table 11). The most connected fungal nodes to chemical compositions belonged to the fungal class Sordariomycetes, Eurotiomycetes, and Dothideomycetes (Supplemental Fig. 9.B). Among these connected microbial nodes to chemical compounds, we were able to identify the significantly positively and negatively associated bacterial (Fig. 6A) and fungal class (Fig. 6B) and genus (Supplemental Fig. 10) to each chemical class. Some microbial class exhibited consistent negative relationships with chemical compounds such as members of the Actinobacteria and Vicinamibacteria class, while others had a positive relationship such as Phycisphareae, Spirochaetia, Mucoromycetes and Umbelopsidomycetes. When focusing on chromone derivative, we found 10 bacterial and 5 fungal class positively associated with increased chromone derivative proportion, and 9 bacterial and 2 fungal class negatively associated. For example, Dothideomycetes and Sordariomycetes were negatively associated with chromone and possible chromone derivative proportion in the black wood. These relationships are correlative and do not demonstrate causal or functional interactions between microbial taxa and chemical compounds, but rather highlight co-occurrence patterns at the OTU/ASV and chemical compound level that may guide future targeted investigations.

## Discussion

### French Guiana, a Suitable Terroir For Quality Agarwood

Endophytic microbial communities of French Guiana *Aquilaria* resemble those of South-East Asia, where *Bacillus* and *Pantoea* were also reported [40], as well as *Aspergillus*, *Penicillium* or *Fusarium* [11]. Numerous saprotrophs and pathotrophs fungi were identified in black wood. For example, *Trechispora*, highly abundant in black wood, is a white rot commonly found on decaying wood [41]. *Botryosphaeriaceae* were necrotrophic or saprobic in woody tissues and were also found in high abundance in black wood. *Calosphaeriaceae*, *Oliveonia*, *Coniochaetaceae*, *Helotiaceae*, *Kylindria*, *Stypella* were among the most commonly found wood saprobes in black wood. Some fungi may also exhibit a necrotic phase such as the microfungus belonging to *Glomerellaceae* family*.*

Although overall microbial community composition was conserved across ampling sites, location‑specific differences were observed. Spatial processes have been show to impact the fungal communities of grapevine, where distance decay relationships of microbial community dissimilarity were found on small distances [42]. In our case, the differences in soil composition, where the plot C had more iron and chromium content, may have impacted microbial communities, and thus, agarwood chemical composition. The specificity of Guianese agarwood is further supported by the strong chemical composition differences we found between local and foreign samples (Supplemental Fig. 5). Plant genotype, micro-climatic environment and agricultural practices may also drive microbial differences in soil and wood endophytic communities, as demonstrated in the vineyards [15, 43]. This is supported by the specific microbial taxa we found between plots, where geographical distance had an impact on soil chemistry and microclimatic conditions, probably caused by contrasting microclimatic conditions, and thus, microbial communities. However, the extent to which specific microbial communities drive desirable chemical terroirs and sensory outcomes remains to be determined. While it was not investigated here, the specific microbiome and climate of French Guiana might also influence the maceration process, and further increase the specificity of the resulting essential oil following distillation.

While *Aquilaria* show promises in French Guiana, it originates from South-East Asia, and its implantation should follow strict environmental precautions. *Aquilaria* spp. can grow from 15 to 25 m in height and have a low number of flowers per inflorescence, obligately outcrossing and the barochoric dispersal of seed do not spread far from the trunk [44]. These trait with the lifespan of the seeds of only a few days, may limit *Aquilaria* invasiveness in French Guiana [45] but careful monitoring following its plantation should be implemented. No report of invasiveness has been identified as of today, whether within the natural distribution area of *Aquilaria* spp. or from plantations within and outside the natural distribution area.

### The Interplay of Plant and its Microbiome for Agarwood Scent

It remains difficult to assess a direct link between chemical compounds and microbial composition. For example, while bacterial and fungal taxa were positively linked to chromone derivative abundance, some biosynthesis pathways exclusively pertain to the plant such as the aromatic ketone benzylacetone or 4-phenylbutan-2-one. This secondary metabolite, present in flowers is an attractant for pollinators [46] and the acyltransferase capable of synthesizing benzylbenzoate [47] is only found in *Magnolopsida* and *Pinopsida*.

The volatile organic compounds belonging to sesquiterpenes are produced by both plant and fungi to attract insect pollinators, defend against pathogens and modulate microbial interactions with antagonistic or synergistic effects on other microbial organisms [48]. Among the fungal kingdom, Basidiomycota are known to produce sesquiterpenes via humulane [49], and Ascomycota fungi also produce volatile sesquiterpenes such as members of the genus *Penicillium*. In our study, we found the *Penicillium* genus to be strongly positively associated at the ASV level with chromone derivatives and sesquiterpenoid eudesmane compounds. While this association does not demonstrate a causal role, it suggests that Penicillium spp. may be involved in biochemical processes linked to agarwood formation and could represent a promising candidate for future experimental investigation. Other genera, including *Rhizobium*, *Solirubrobacter*, *Acrocalymma* and *Curvularia*, also showed significant associations with agarwood chemical composition, highlighting their potential relevance in shaping wood chemical profiles. These taxa may be linked to a higher concentration of odorant molecules either through their direct biosynthesis, inducing stronger plant defenses, or modulating microbial competition. Further analysis on the effect of these genera on plant fitness and stress response could provide valuable insights in the production of quality agarwood. Methods such as synthetic communities could be used to produce an optimal inoculum, boosting plant growth and inducing plant response following stress [50]. For the first time, we provide an in-depth analysis of bacterial and fungal taxa associated with volatile constituents of agarwood, and identified positively and negatively associated microbial members to each chemical class. The positively associated taxa with chromone derivative such as members of the Alphaproteobacteria, Baccilli, Thermophilia, Verrucomicrobiia, Agaromycetes, or Mucoromycetes could provide valuable local inoculum resource, for tree infection or maceration starter. Further studies should test the effect of complex communities’ inoculation on agarwood formation throughout plant development and their involvement in the metabolomic induction of biosynthesis pathways relevant to agarwood scent.

## Conclusion

This study provides a comprehensive in-depth characterization of the bacterial and fungal communities associated with agarwood produced from *A. crassna* cultivated in French Guiana and examines their relationships with volatile chemical constituents relevant to agarwood scent. Our results demonstrate that *Aquilaria* grown outside its native range in French Guiana is capable of producing high‑quality agarwood, with a conserved chemical profile across distinct sampling sites, supporting the existence of a stable chemical terroir under local environmental conditions. By integrating microbial community profiling with detailed chemical analyses, we identified numerous bacterial and fungal taxa that were positively or negatively associated with major chemical classes, including chromone derivatives and sesquiterpenoids, which are key contributors to agarwood fragrance. Although these relationships remain correlational, they suggest that microbial communities may influence agarwood chemical complexity through interactions with plant stress responses, microbial metabolism, or cross‑kingdom ecological processes. The persistence of a conserved chemical profile despite spatial variability in microbial composition further indicates potential functional redundancy among microbial taxa or a strong host‑mediated regulation of secondary metabolite production. Comparisons with commercial agarwood samples from South-East Asia in the Middle East revealed a distinct chemical signature of Guianese agarwood, reinforcing the influence of geographic origin on agarwood quality and supporting an extension of the terroir concept to woody aromatic products. However, the uncertain history of commercial samples, including potential effects of storage, transport, and handling, should be considered when interpreting these differences. These findings highlight the importance of considering both microbial communities and environmental context when evaluating agarwood quality and origin. Overall, this work provides a foundation for the development of microbiome‑informed strategies for agarwood induction, maceration, and processing based on local microbial resources. Future research combining experimental inoculation, longitudinal sampling, and metabolic approaches will be essential to disentangle causal relationships between microbial taxa and chemical traits and to optimize sustainable agarwood production.

## Supplementary Information

Below is the link to the electronic supplementary material.

Supplementary file1 (PPTX 11361 KB)

Supplementary file2 (XLSX 552 KB)

Supplementary file3 (XLSX 191 KB)

Supplementary file4 (XLSX 127 KB)

Supplementary file5 (XLSX 53 KB)

Supplementary file6 (XLSX 11 KB)

Supplementary file7 (XLSX 97 KB)

Supplementary file8 (XLSX 14 KB)

Supplementary file9 (XLSX 63 KB)

Supplementary file10 (XLSX 79 KB)

Supplementary file11 (XLSX 15 KB)

Supplementary file12 (XLSX 78 KB

## Data Availability

Sequencing data are available at the NCBI database (Bio-project PRJNA1249911, Biosamples SAMN47917871 to SAMN47917976). All metadata are provided as supplementary tables.

## Code Availability

The datasets generated during and/or analyzed during the current study are provided in supplementary tables and the code is available from the corresponding author on reasonable request.
